# High central venous oxygen saturation in the latter stages of septic shock is associated with increased mortality

**DOI:** 10.1186/cc10325

**Published:** 2011-07-26

**Authors:** Julien Textoris, Louis Fouché, Sandrine Wiramus, François Antonini, Sowita Tho, Claude Martin, Marc Leone

**Affiliations:** 1 Service d'anesthésie et de réanimation. Hôpital Nord, Assistance Publique - Hôpitaux de Marseille, Université de la Méditerranée, Chemin des bourrely, 13915, Marseille Cedex 20, France

## Abstract

**Introduction:**

Current guidelines recommend maintaining central venous oxygen saturation (ScvO_2_) higher than 70% in patients with severe sepsis and septic shock. As high levels of ScvO_2 _may reflect an inadequate use of oxygen, our aim was to evaluate the relation between maximal ScvO_2 _levels (ScvO_2*max*_) and survival among intensive care unit (ICU) patients with septic shock.

**Methods:**

We retrospectively analyzed data from all admissions to our ICU between January 2008 and December 2009. All septic shock patients in whom the ScvO_2 _was measured were included. The measures of ScvO_2*max *_within the first 72 hours after the onset of shock were collected.

**Results:**

A total of 1,976 patients were screened and 152 (7.7%) patients met the inclusion criteria. The level of ScvO_2*max *_was 85% (78 to 89) in the non-survivors, compared with 79% (72 to 87) in the survivors (*P *= 0.009).

**Conclusions:**

Our findings raise concerns about high levels of ScvO_2 _in patients with septic shock. This may reflect the severity of the shock with an impaired oxygen use. Future strategies may target an optimization of tissue perfusion in this specific subgroup of patients.

## Introduction

Shock is characterized by either an inadequacy between tissue requirements in oxygen and oxygen delivery or the inadequate use of oxygen. The hemodynamic management of patients in shock aims at improving tissue oxygenation. Central venous blood saturation in oxygen (ScvO_2_) is a useful tool reflecting the global transport and metabolism of oxygen. International guidelines suggest the need to optimize ScvO_2 _in the early phase of management of severe sepsis and septic shock [1].

Low levels reflect (i) an inadequate cardiac output with an excessive extraction of oxygen [2], (ii) a low hemoglobin concentration, and/or (iii) a low level of arterial oxygen pressure (PaO_2_). In contrast, high levels of ScvO_2 _means either (i) a very high oxygen delivery in excess of tissue requirements and/or (ii) decreased cellular consumption of oxygen (mitochondrial dysfunction) and/or (iii) more rarely, a large arterio-venous shunt. In its most simple form, the oxyhemoglobin dissociation curve describes the relation between the partial pressure of oxygen (x axis) and the oxygen saturation (y axis). Many factors influence the affinity of this binding, altering the curve shape. For example, acidosis and body hyperthermia induce a right shift of the curve. This shift promotes the release of oxygen to the tissues.

Because high levels of ScvO_2 _could reflect an impaired extraction of oxygen, our hypothesis was that ScvO_2 _levels above 80% in patients in septic shock hospitalized in ICU should be correlated with an increased mortality rate. The present study was aimed to correlate high levels of ScvO_2 _with mortality.

## Materials and methods

Approval by the Ethics Committee and informed consent were waived due to the observational nature of the study. From January 2008 to December 2009, the charts of all patients admitted to the ICU of our institution (Hôpital Nord, Marseilles, France) were retrospectively screened. We included patients aged ≥ 18 yrs in septic shock, as defined elsewhere [3], and at least two available ScvO_2 _samples within the first 72 hours of shock. The patients with an ICU stay < 24 hours were excluded.

The central venous catheter sampling site was in the superior vena cava. The position of the tip of the catheter was checked with chest radiography. Within the first 72 hours of shock, we collected the maximal ScvO_2 _(ScvO_2*max*_), and minimal ScvO_2 _(ScvO_2*min*_) levels. The characteristics of patients were also collected, including: demographics (age, sex, body mass index (BMI), admission simplified acute physiology score (SAPS) II [4], and sequential organ failure assessment (SOFA) score (measured at the onset of shock)) [5]. Variables such as plasma lactate level, plasma creatinine level, daily urine output, ratio of arterial oxygen pressure related to inspired oxygen fraction (PaO_2_/FiO_2_), hemoglobin, heart rate, mean arterial pressure, pulsatile saturation in oxygen (SpO_2_) and body temperature were recorded contemporary to the ScvO_2*max *_value. The patients were stratified according to their hospital mortality. The patients in septic shock were managed according to our local protocol in agreement with the Surviving Sepsis Campaign guidelines [1]. Briefly, fluid resuscitation was conducted according to dynamic indices of preload (pulse pressure variations and stroke volume variations) and echocardiography data analysis. Norepinephrine was used as the first line vasopressor in order to achieve a mean arterial pressure of 65 mmHg. As reported elsewhere, positive inotropes (dobutamine or isoproterenol) were introduced in the patients with low cardiac index and ScvO_2 _< 70% [1,6]. Renal replacement therapy was used only in patients exhibiting anuria or elevated potassium levels.

Statistical calculations were performed using the R project software (version 2.10). For continuous and ordinal variables, data were expressed as median with interquartile range. Comparisons between the two groups (survivors and non-survivors) were performed for epidemiological data and for hemodynamic data using a Mann-Whitney U test. For dichotomous variables, percentages were computed. Comparisons of percentages were performed with the Chi² test. We also performed a multivariate analysis by logistic regression to control for potential confounders in evaluating the relationship between ScvO_2*max *_and outcome. Variables introduced into the model were those found to be significant in univariate analysis (with the exception of creatinine level, known to be linked to daily urine output, and SAPS II): body temperature, daily urine output, ScvO_2*max*_, and plasma lactate. We checked the prediction models' residuals were distributed normally. All comparisons were two-tailed, and *P = *0.05 was required to exclude the null hypothesis.

## Results

**Figure 1:**
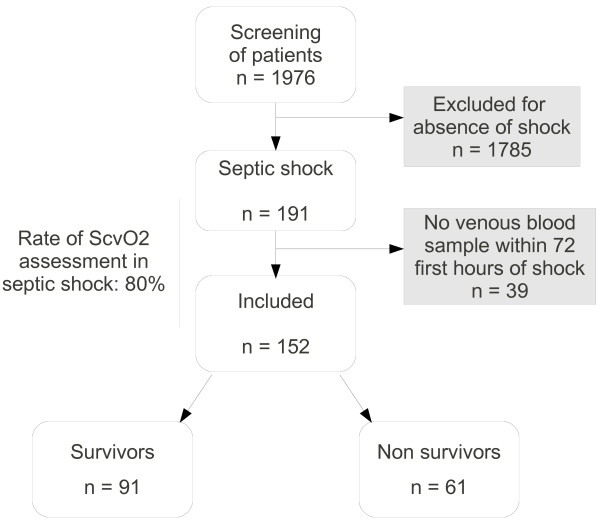
**Flowchart of the study**.

The screening of 1,976 charts identified 152 (7.7%) patients in septic shock fulfilling the inclusion criteria (Figure 1). All the patients received sedation (Ramsay score between 3 and 5) and were ventilated. Norepinephrine was used in all patients. Thirty-four (22%) patients were treated by positive inotrope. Ninety-one (60%) patients were discharged from the hospital. At admission, the SAPS II score was significantly higher in the non-survivors than in the survivors (58 (38 to 72) *vs*. 44 (33 to 55); *P = *0.01). At the onset of septic shock, the SOFA score did not differ between the survivors and the non-survivors. The body temperature and the daily urine output were higher in the survivors than in the non-survivors (38.1°C (37.3 to 39) *vs *37.2°C (36.5 to 38), *P *= 0.00004, and 1,200 mL/day (700 to 1,600) *vs *685 mL/day (415 to 1,425), *P *= 0.006, respectively). The plasma creatinine level and the plasma lactate level were lower in the survivors than in the non-survivors (102 μmol/L (73 to 161) *vs *186 μmol/L (98 to 280), *P *= 0.0002, and 2.1 mmol/L (1.5 to 2.9) *vs *2.4 mmol/L (1.8 to 3.7), *P = *0.05, respectively) (Table 1).

**Table 1:** Results of univariate analysis

	All patients *n *= 152	Survivors *n *= 91	Non-survivors *n *= 61	*P*
**Sex female**	51 (34)	31 (34)	20 (33)	0.99
**Age (yrs)**	65 (53 to 74)	66 (52 to 74)	64 (57 to 75)	0.29
**Body mass index**	24 (22 to 28)	24 (22 to 28)	24 (22 to 29)	0.77
**Body temperature (°C)**	38.0 (37.0 to 38.5)	38.1 (37.3 to 39.0)	37.2 (36.5 to 38.1)	0.00004*
**ScvO_2*min *_(%)**	68 (62 to 73)	69 (63 to 73)	68 (60 to 73)	0.61
**ScvO_2*max *_(%)**	83 (74 to 88)	79 (72 to 87)	85 (78 to 89)	0.009*
**Heart rate (beats/minute)**	105 (93 to 120)	104 (92 to 117)	108 (94 to 127)	0.21
**Mean arterial pressure (mmHg)**	71 (62 to 81)	74 (63 to 77)	68 (61 to 81)	0.07
**SpO_2 _(%)**	96 (93 to 100)	96 (94 to 100)	96 (92 to 98)	0.16
**Glasgow Coma Scale**	15 (12 to 15)	15 (13 to 15)	13 (7 to 15)	0.09
**Urine output (mL/day)**	1,100 (600 to 1,600)	1,200 (700 to 1,600)	685 (415 to 1,425)	0.006*
**Creatinine (μmol/L)**	119 (78 to 204)	102 (73 to 161)	186 (98 to 280)	0.0002*
**PaO_2_/FiO_2_**	280 (123 to 365)	321 (128 to 380)	184 (131 to 202)	0.16
**Hemoglobin (g/dL)**	9.5(8.5 to 11.0)	9.7 (8.5 to 10.9)	9.4 (8.4 to 10.6)	0.51
**pH** **Lactate (mmol/L)**	7.35 (7.27 to 7.41) 2.1 (1.6 to 3.4)	7.37 (7.32 to 7.41) 2.1 (1.5 to 2.9)	7.33 (7.21 to 7.41) 2.4 (1.8 to 3.7)	0.11 0.05*
**ICU stay (days)**	8 (4 to 19)	8 (4 to 18)	8 (4 to 20)	0.93
**SAPS 2**	47 (37 to 63)	44 (33 to 55)	58 (38 to 72)	0.01
**SOFA**	7 (5 to 11)	7 (3 to 10)	8 (5 to 11)	0.40

ICU, intensive care unit; PaO_2_/FiO_2 _ratio, ratio of arterial oxygen pressure related to inspired oxygen fraction; SAPS, simplified acute physiologic score; SpO_2_, pulse oxymetry; SvcO_2_, central venous oxygen saturation; SOFA, Sequential Organ Failure Assessment score.

*variable included in the multivariate analysis. Results are expressed as number (percentage) or median (Interquartile range), as required.

**Figure 2:**
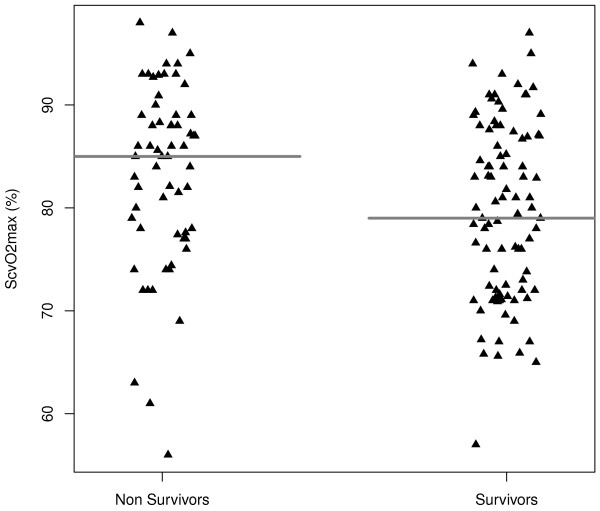
**Maximal central venous oxygen saturation (ScvO_2*max*_) is higher in the non-survivors than in the survivors**.

The ScvO_2*max *_was 83% (74 to 88%) and the median ScvO_2*min *_was 68% (62 to 73%). Minimal ScvO_2 _was not different between the survivors and the non-survivors (Table 1). In contrast, the ScvO_2*max *_was of 79% (72 to 87%) in the survivors, as compared with 85% (78 to 89%) in the non-survivors (*P *= 0.009) (Figure 2). In these patients, only a high level of ScvO_2*max *_(OR = 1.06; CI 95% = (1.01 to 1.13)) and a lower body temperature (OR = 0.48; CI 95% = (0.32 to 0.70)) were associated with hospital mortality. A contingency table with a cut-off of ScvO_2*max *_at 80% is shown in Table 2. According to this cut-off, the rates of mortality were 30% (ScvO_2*max *_< 80%) and 48% (ScvO_2*max *_≥ 80%), respectively (*P *= 0.04).

**Table 2:** Contingency table of mortality among a ScvO_2*max *_cut-off of 80%

		**Non survivors**	**Survivors**	**Mortality (%)**	***P*-value**
		
ScvO_2*max*_	< 80%	20	46	30	0.04
	≥ 80%	41	45	48	

## Discussion

In the present study, the ScvO_2*min *_cannot serve to discriminate the survivors and the non-survivors, and high ScvO_2*max *_levels are associated with increased mortality. Thus, a dysoxia with an impaired extraction of oxygen may be suspected in septic shock patients.

The ScvO_2*min *_was not associated with increased mortality. At first glance, this finding can be surprising. In the emergency department (ED), an early correction of ScvO_2 _was related to improved outcome [7]. In the ICU, the baseline values of SvO_2 _(69% [8]) seem to be higher than that observed in the ED (49% [7]). Thus, although an aggressive resuscitation of the subgroup of patients with ScvO_2 _below 70% may be beneficial, these low levels are less frequently observed in ICU patients than in ED patients. Low ScvO_2 _levels may be due to a combination of enhanced oxygen consumption, inadequately low cardiac index, low hemoglobin concentrations, and/or low arterial oxygen content. In the present study, all patients had endotracheal intubation and were ventilated. In addition, they were sedated by a combination of sufentanil and midazolam. Probably both of these treatments minimized the oxygen consumption [9]. In addition, the mean SpO_2 _was 96%, and the mean level of hemoglobin at 9.5 g/dL. Thus, in the majority of our patients, a low level of ScvO_2 _may only reflect an inadequately low cardiac index [10]. This particular case is infrequent in adequately resuscitated septic shock patients [11].

One of the objectives of septic shock resuscitation is to raise the ScvO_2 _level above 70%. Our study underlines that in patients with organ failure, high ScvO_2 _values may be a marker of unfavorable outcome. This result may not be generalizable to all septic patients. All our patients were sedated and mechanically ventilated. A previous study showed that SvcO_2 _increases significantly in response to intubation [9]. In addition, the ScvO_2 _values were obtained within the first 72 hours of ICU stay. This differs from a previous study, which was performed after the patient admission in the ED [7]. Future strategies should include subgroup stratification based on the ScvO_2 _levels. Such a model was suggested for patients admitted to the ED [12]. In this study, ScvO_2 _levels were stratified into three groups: hypoxia (ScvO_2 _< 70%); normoxia (ScvO_2 _71% to 89%); and hyperoxia (ScvO_2 _90% to 100%). Using a multivariate analysis, the authors showed that hyperoxia was associated with increased mortality. Similar results were reported after cardiac surgery [13].

This clinical finding supports the hypothesis of a decreased use of oxygen in sepsis [14]. An acquired defect in oxidative phosphorylation prevents cells from using molecular oxygen for adenosine triphosphate production and potentially causes sepsis-induced organ dysfunction. This misuse of oxygen, so-called cytopathic dysoxia, can result in high levels of ScvO_2_. In our study, we used an arbitrary cut-off at 80%. The difference in mortality between the patients with ScvO_2*max *_value below or above 80% may generate clues regarding specific therapeutic strategies such as administering ubiquinone derivative targeted towards mitochondria in septic patients with dysoxia [15].

One may suspect microcirculatory impairments in patients with high ScvO_2 _levels. In line with this hypothesis, plasma lactate levels were higher in the non-survivors than in the survivors. Nevertheless, we did not find that ScvO_2*max *_levels and plasma lactate levels were correlated (data not shown, assessed by a generalized linear model, *P *= 0.62, r² = 0.14). The rise of plasma lactate level in shock is not only due to tissue hypoxia, but it can also be the consequence of the muscular aerobic Na^+^/K^+ ^ATP_ase _driven production in response to an intense catecholaminergic stimulation [16]. This second mechanism may explain the absence of correlation. The microcirculation remains to be evaluated in septic patients with dysoxia. In a prior study, we showed that low tissue oxygen saturation (StO_2_) was associated with increased mortality [17]. It would be interesting to investigate specifically the microcirculation of patients with high levels of ScvO_2_.

The present study has several limitations. As this single-center study is retrospective, a selection bias might be possible. In our protocol, we did not measure ScvO_2 _continuously, thus we might have missed several events. We did not measure oxygen consumption, which may be important for the interpretation of this finding. Moreover, as we collected ScvO_2 _during the first 72 hours of resuscitation, ScvO_2 _measurements may reflect various states of the treatment. One can suggest that the arterial oxygen saturation may influence the ScvO_2 _levels. However, this variable did not differ between survivors and non-survivors. Another selection bias might be that patients with a low venous oxygen saturation are more likely to die in the first 48 hours after the onset of septic shock. However, in our study, only nine patients (6%) died within this period. Their mean ScvO_2*min *_levels were 68%. As we stated in the methods section, our protocol includes a preload assessment to guide fluid and global hemodynamic management of patients. At the bedside, the cardiac output was continuously monitored in more than 60% of our patients. Unfortunately, these data were not systematically reported in our patients' charts. More than 10% of data was lacking, therefore rendering the analysis difficult. Norepinephrine was used in all our patients to achieve a mean arterial pressure of 65 mmHg. The restoration of hemodynamics by using vasopressors may cause venous hyperoxia, by increasing cardiac output. As the use of high doses of vasopressors has been associated with increased mortality [18], this may be another potential confounding factor.

## Conclusions

In conclusion, high levels of ScvO_2 _in septic shock patients may be associated with increased mortality. Future studies relating outcome and ScvO_2 _should stratify the patients according to venous hypoxia, normoxia and hyperoxia levels.

## Key messages

• High levels of ScvO_2 _(> 80%) during the first 72 hours of resuscitation of septic shock patients is associated with increased mortality

• High levels of ScvO_2 _could reflect an impaired use of oxygen

## Abbreviations

BMI: Body Mass Index; ED: emergency department; FiO_2_: inspired fraction in oxygen; CI: confidence interval; ICU: intensive care unit; OR: odds ratio; PaO_2_: arterial pressure of oxygen; SAPS II: Simplified Acute Physiology Score II; ScvO_2_: central venous oxygen saturation; SOFA: Sequential Organ Failure Assessment; SpO_2_: pulsatile oxygen saturation; StO_2_: tissular oxygen saturation.

## Competing interests

The authors declare that they have no competing interests.

## Authors' contributions

All the authors contributed to the elaboration of this manuscript. LF and ST performed data extraction and wrote the manuscript draft. JT and SW performed data management and wrote the manuscript. FA performed data management and statistical analysis, while CM and ML did the study conception and wrote the manuscript. All authors read and approved the final version of the manuscript.
